# Identification of New *Mycobacterium tuberculosis* Proteasome Inhibitors Using a Knowledge-Based Computational Screening Approach

**DOI:** 10.3390/molecules26082326

**Published:** 2021-04-16

**Authors:** Tahani M. Almeleebia, Mesfer Al Shahrani, Mohammad Y. Alshahrani, Irfan Ahmad, Abdullah M. Alkahtani, Md Jahoor Alam, Mohd Adnan Kausar, Amir Saeed, Mohd Saeed, Sana Iram

**Affiliations:** 1 Department of Clinical Pharmacy, College of Pharmacy, King Khalid University, Abha 61421, Saudi Arabia; talmelby@kku.edu.sa; 2 Department of Clinical Laboratory Sciences, College of Applied Medical Sciences, King Khalid University, Abha 61421, Saudi Arabia; mesferm@kku.edu.sa (M.A.S.); moyahya@kku.edu.sa (M.Y.A.); irfancsmmu@gmail.com (I.A.); 3 Research Center for Advanced Materials Science (RCAMS), King Khalid University, Abha 61421, Saudi Arabia; 4 Department of Microbiology and Clinical Parasitology, College of Medicine, King Khalid University, Abha 61421, Saudi Arabia; abdalqahtani@kku.edu.sa; 5 Department of Biology, College of Sciences, University of Hail, Hail 2240, Saudi Arabia; j.alam@uoh.edu.sa (M.J.A.); mo.saeed@uoh.edu.sa (M.S.); 6 Department of Biochemistry, College of Medicine, University of Hail, Hail 2240, Saudi Arabia; ma.kausar@uoh.edu.sa; 7 Department of Clinical Laboratory Sciences, College of Applied Medical Sciences, University of Hail, Hail 2240, Saudi Arabia; am.saeed@uoh.edu.sa; 8 Faculty of Medical Laboratory Sciences, Department of Medical Microbiology, University of Medical Sciences & Technology, Khartoum 12810, Sudan; 9 Department of Medical Biotechnology, Yeungnam University, Gyeongsan 38541, Korea; 10 Nanomedicine & Nanobiotechnology Laboratory, Department of Biosciences, Integral University, Lucknow 226026, India

**Keywords:** *Mycobacterium tuberculosis*, tuberculosis, proteasome, natural compounds

## Abstract

*Mycobacterium tuberculosis* (Mtb) is a deadly tuberculosis (TB)-causing pathogen. The proteasome is vital to the survival of Mtb and is therefore validated as a potential target for anti-TB therapy. Mtb resistance to existing antibacterial agents has enhanced drastically, becoming a worldwide health issue. Therefore, new potential therapeutic agents need to be developed that can overcome the complications of TB. With this purpose, in the present study, 224,205 natural compounds from the ZINC database have been screened against the catalytic site of Mtb proteasome by the computational approach. The best scoring hits, ZINC3875469, ZINC4076131, and ZINC1883067, demonstrated robust interaction with Mtb proteasome with binding energy values of −7.19, −7.95, and −7.21 kcal/mol for the monomer (K-chain) and −8.05, −9.10, and −7.07 kcal/mol for the dimer (both K and L chains) of the beta subunit, which is relatively higher than that of reference compound HT1171 (−5.83 kcal/mol (monomer) and −5.97 kcal/mol (dimer)). In-depth molecular docking of top-scoring compounds with Mtb proteasome reveals that amino acid residues Thr1, Arg19, Ser20, Thr21, Gln22, Gly23, Asn24, Lys33, Gly47, Asp124, Ala126, Trp129, and Ala180 are crucial in binding. Furthermore, a molecular dynamics study showed steady-state interaction of hit compounds with Mtb proteasome. Computational prediction of physicochemical property assessment showed that these hits are non-toxic and possess good drug-likeness properties. This study proposed that these compounds could be utilized as potential inhibitors of Mtb proteasome to combat TB infection. However, there is a need for further bench work experiments for their validation as inhibitors of Mtb proteasome.

## 1. Introduction

*Mycobacterium tuberculosis* (Mtb) is a deadly tuberculosis (TB)-causing pathogen. TB is a communicable disease that ranks in the world’s top 10 causes of death. Besides, it is the leading cause of single infectious agent fatality (higher than HIV/AIDS), and approximately 10 million people fell ill with TB in 2019 [1]. A person with a weakened immune system is highly susceptible to TB infection; thus, their involvement with HIV is the major cause of fatality for these patients [2]. Mtb resistance to existing antibacterial agents has enhanced drastically as well as multidrug-resistant and extensively drug-resistant Mtb strains. These strains are becoming a worldwide health issue [3,4] and are involved in the host immune system’s resistance to nitric oxide stress.

Proteasomes are multi-subunit proteolytic complexes that have a vital role in various cellular functions. Proteasome inhibition has appeared as a prevailing approach for the management of various infectious diseases [5]. Mtb proteasome is vital for the bacterium pathogenesis; hence, it is regarded as an attractive target for the development of new agents that may inhibit Mtb. The Mtb mutans lacking the proteasome (proteasome subunits silencing) are viable in vitro, but the infection cannot be maintained in the TB mouse model [6,7]. Hence, it seems that Mtb proteasomes are vital for their propagation in mammalian hosts and are involved in the host immune system’s resistance to nitric oxide stress [8].

The proteasome is a heap-shaped protein made up of four rings of heptamers. Its length and width are 150 and 115 Å, respectively [9,10]. Inner beta rings are formed by seven identical prcB subunits, and outer alpha rings are formed by seven identical prcA subunits, which give a path to inner beta rings with active sites when they are open. As a result, it provides the overall organization of α7β7β7α7. The active site of the bacterial proteasome is identical to that of the archaeal and eukaryotic proteasomes and is found primarily in β subunits [9,11].

In the literature, several effective Mtb proteasome inhibitors have been documented. Among them, HT1171, GL5, MLN273, and fellutamide-B are the most potent Mtb proteasome inhibitors [9,12,13]. The Mtb proteasome was revealed to be inhibited by 15 psoralens from a library of 92 analogs, and compounds 8, 11, 13, and 15 exhibited potent inhibition in a fluorescence-based enzymatic assay [14]. Several plant-derived natural products were discovered to inhibit Mtb proteasome with IC_50_ values ranging from 25 to 120 M using the chymotrypsin substrate Suc-LLVY-AMC [15]. Various bortezomib analogs have been developed, with the phenol- and halogen-substituted analogs being more specific for the Mtb proteasome than the human proteasome [16].

Drug development requires the identification of some potential hits from a huge library of chemical components. Screening such a huge compounds library using wet-lab assays can be a difficult task. Protein-ligand docking is a powerful tool in drug development because it aids in the identification of active or lead compounds from a library of compounds [17,18]. This method is also capable of accurately identifying inhibitor binding modes to the target proteins [17,19]. Computational screening before laboratory testing is a successful approach in decreasing the number of candidate inhibitors for benchwork-based screening [20,21,22]. The present study aimed to identify new possible hits from the natural compounds databases using in silico, state-of-the-art techniques that could serve as Mtb proteasome inhibitors to combat TB infection.

## 2. Methodology

### 2.1. Protein Structure Preparation

Mtb proteasome 3D structure (PDB ID: 5TRG) was retrieved from the protein data bank and prepared in monomer form by Discovery Studio (DS) 2020. Since the Mtb proteasome core particle has 14 chains in the beta subunit, all of which have the same active sites, the present study focused on chain K as a monomer, and the K and L chains as a dimer.

### 2.2. Database Collection and Refinement

Natural compounds were accessed from the ZINC database (https://zinc.docking.org accessed on 29 January 2021), limiting the outcomes by choosing “natural products” as a subset, resulting in a total of 224,205 compounds and was then downloaded in SDF format. Furthermore, these compounds were processed for minimization and preparation for screening using the “ligand preparation” tool in DS 2020.

### 2.3. Receptor-Based Virtual Screening

In order to identify possible leads, the prepared librarian was screened against the Mtb proteasome active site using AutoDock vina (version 1.1.2). Then, the top-scored hits were further processed for in-depth molecular docking studies.

### 2.4. Molecular Docking

Lead hits were docked with Mtb proteasome (monomer; K-chain) by Autodock4.2 to determine the ligand–protein interaction and their binding affinities [23]. All input files were prepared using AutoDock Tools, adding polar hydrogen in protein, and assigning the charges with the Kollman charges method. The grid center points X, Y, and Z were kept as 36.731, −11.255, and 43.313, respectively. Grid points were fixed as 60 × 60 × 60 Å with the spacing of 0.375 Å. Additionally, these hits were also docked with the dimer form (K and L chains) of the beta subunit of the Mtb proteasome, keeping the grid center points X, Y, and Z as 41.22, 0.60, and 32.31, respectively. All docking calculation parameters were kept as a default value. The highest negative binding energy (BE) value was ranked as the most promising binding pose.

### 2.5. LIGPLOT^+^ Analysis

The H-bond and hydrophobic interactions between “hit compounds–Mtb proteasome” complexes were determined by the LIGPLOT+ Version v.2.1. The 3-D structures of the “compound–Mtb proteasome” interaction produced were transformed into 2-D figures using the LIGPLOT algorithm.

### 2.6. Drug-Likeness

Top-scoring molecules (ZINC3875469, ZINC4076131, and ZINC1883067) were further used to estimate drug-likeness, toxicity, and pharmacokinetic properties using the pkCSM and SwissADME tools [24]. SMILE IDs of the molecules retrieved from the ZINC database were entered into the pkCSM tool to evaluate drug-likeness [25].

### 2.7. Molecular Dynamics (MD) Simulations

To study the dynamic behavior of the protein–ligand complex in simulated physiological conditions, MD simulations were performed using Gromacs Ver. 2020.4. The protein–ligand complexes were solvated in a 10 × 10 × 10 Å orthorhombic periodic box built with TIP3P water molecules. By adding a sufficient number of 9 Na counterions, the entire system was neutralized. This solvated system was energy-minimized and position-restrained with CHARMM 36 as a force-field [26]. Further, 20 ns of MD run was carried out at 1 atm pressure and 300 K temperature implementing NPT ensemble with a recording interval of 100 ps. This resulted in a total of 1000 reading frames. In the end, various parameters of MD simulation study such as ligand binding site analysis, root-mean-square deviation (RMSD), root-mean-square fluctuation (RMSF), radius of gyration, Mindistance, H-bond analysis, etc., were analyzed for the stability, compactness, structural variations, and protein–ligand interactions in the solvated system.

## 3. Results and Discussion

### 3.1. Virtual Screening, Molecular Docking, and LIGPLOT

**Figure 1:**
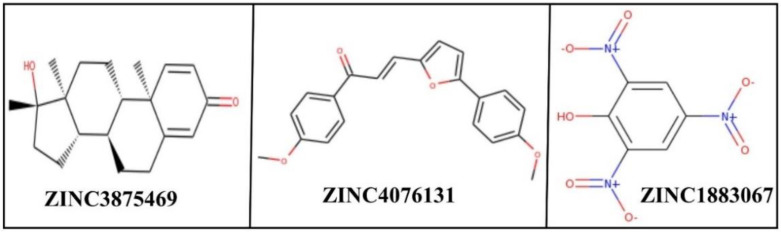
2D structures of hit compounds.

**Figure 2:**
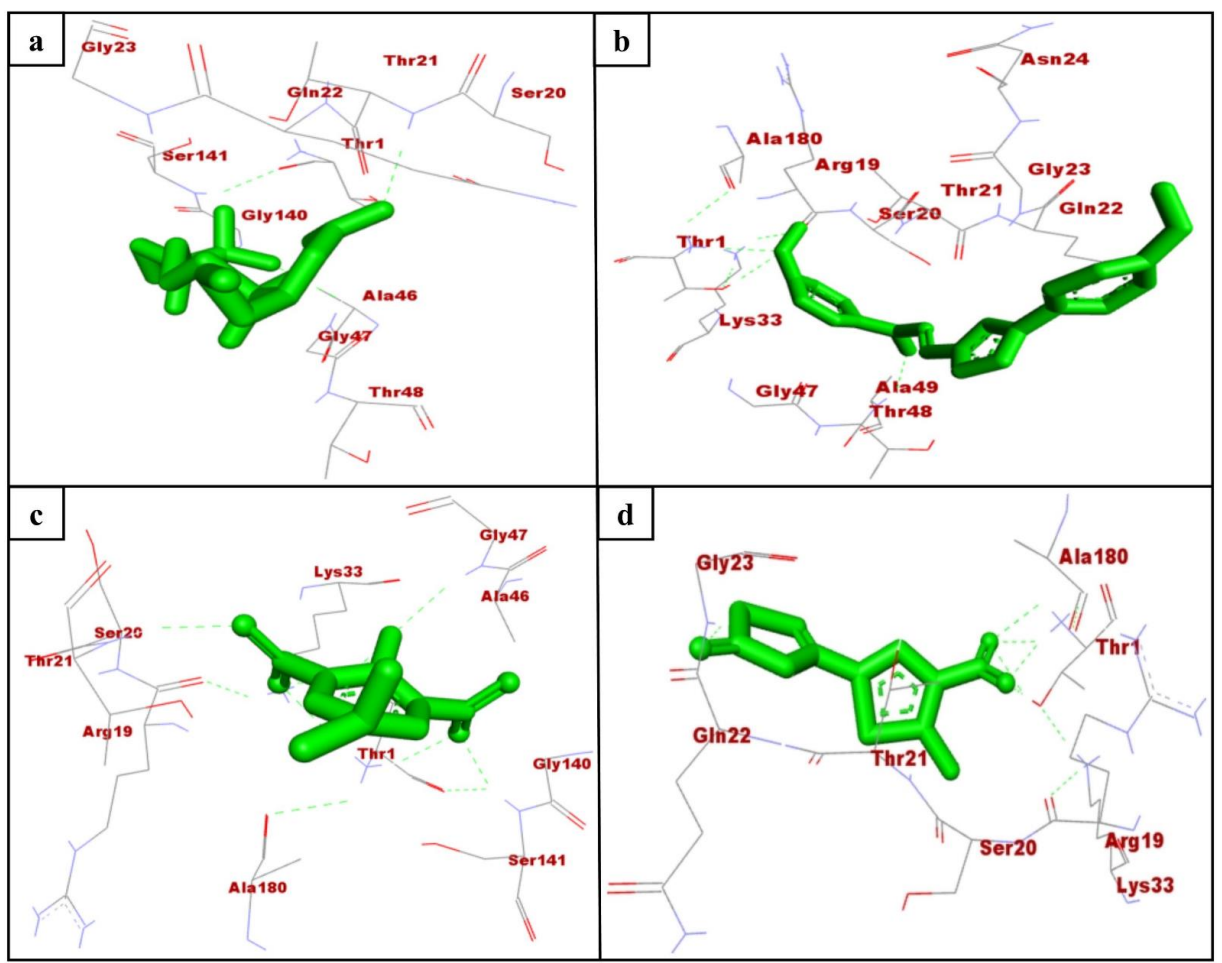
Interacting residues of Mtb proteasome (monomer; K-chain) with ZINC3875469 (**a**), ZINC4076131 (**b**), ZINC1883067 (**c**), and HT1171 (**d**).

Mtb is the only known bacterial pathogen that has proteasome activity [6]. The increase in drug-resistant TB is a major public health concern and requires the development of new agents that can evade the resistance and effectively control TB. With this purpose, we conducted the computational screening of 224,205 natural compounds from the ZINC database targeting the Mtb proteasome. Among them, the selected hits ZINC3875469, ZINC4076131, and ZINC1883067 showed strong binding with the Mtb proteasome. Two-dimensional structures of hit compounds are shown in Figure 1. ZINC3875469 interacted with proteasome through 10 amino acid residues: Thr1, Ser20, Thr21, Gln22, Gly23, Ala46, Gly47, Thr48, Gly140, and Ser141 (Figure 2a); while Thr1, Arg19, Ser20, Asn24, Thr21, Gln22, Gly23, Asn24, Lys33, Gly47, Ala49, and Ala180 residues of the proteasome were observed to bind with ZINC4076131 (Figure 2b). In a similar way, ZINC1883067 was found to interact with Thr1, Arg19, Ser20, Thr21, Lys33, Ala46, Gly47, Gly140, Ser141, and Ala180 residues of the proteasome (Figure 2c).

The active site pocket residues of Mtb proteasome were determined as Ser20, Thr21, Gln22, Val31, Ile45, Ala46, Thr48, Ala49, Val53, Asp124, Asp128, and Asp130 [27]. Interestingly, ZINC3875469, ZINC4076131, and ZINC1883067 were also found to bind with these residues of Mtb proteasome. In a study, small molecules were reported to interact with Thr1, Arg19, Ser20, Thr21, Gln22, Lys33, Gly47, Ala49, Gly140, and Ser141 residues of Mtb proteasome [27]. Consistent with this, in the present study, the selected hits were observed to bind with the similar residues of Mtb proteasome.

Oxathiazol-2-one compounds altered the Mtb proteasome by interacting with the Thr1 residues of the core complex beta-subunit. Consequently, Thr1 is cyclocarbonylated, which greatly alters the active site environment and causes an alternative protein conformation in which the substrate-binding pocket is disrupted. As a result, Mtb proteasome substrates were unable to obtain access to the proteasome, causing toxic proteins and peptides to accumulate within mycobacterial cells. Notably, the oxathiazol-2-one compounds were thought to have no effect on the substrate-binding pocket of human proteasomal beta-subunits. It was proposed that this was due to the fact that the residues involved in preserving the altered conformation were largely non-conserved and thus not susceptible to cyclocarbonylation inactivation. This provided highly selective inhibition of Mtb proteasomes while leaving host proteasomal activity unaffected [13]. Interestingly, in this study, the selected hits ZINC3875469, ZINC4076131, and ZINC1883067 were found to interact with Thr1 residues of the Mtb proteasome.

**Figure 3:**
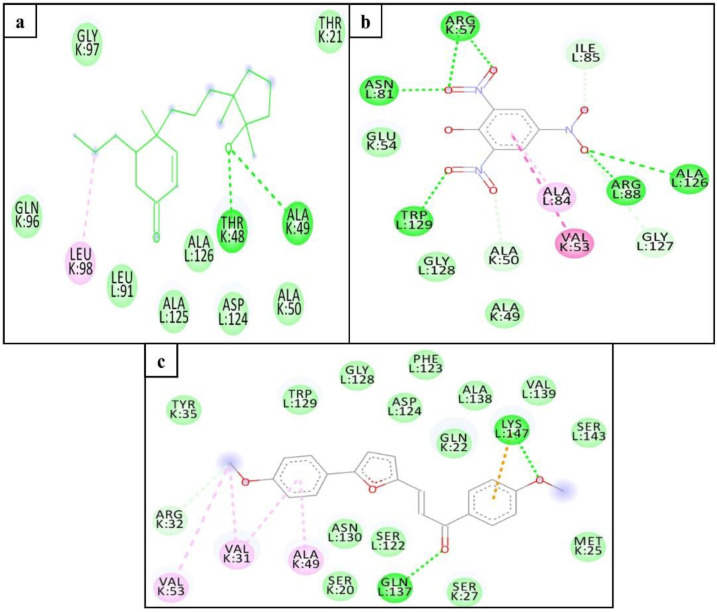
Interacting residues of Mtb proteasome (dimer; K and L chains) with ZINC3875469 (**a**), ZINC1883067 (**b**), and ZINC4076131 (**c**).

We also performed the molecular docking of selected hits with the dimer model (K and L chains) of the Mtb proteasome to determine possible interactions with the adjacent chain (L-chain). Asp124 of the adjacent chain of the Mtb proteasome has been reported as an important residue for inhibitor interaction [28]. Interestingly, in addition to interacting with K-chain residues, ZINC3875469 and ZINC4076131 interacted with several other Mtb proteasome L-chain residues including the Asp124 (Figure 3a,c). Despite the fact that ZINC1883067 did not interact with the Asp124 residue of the L- chain, it did interact with several other residues of both chains of the Mtb proteasome (Figure 3b).

The BE values for hits ZINC3875469, ZINC4076131, and ZINC1883067 with the Mtb proteasome were found to be −7.19, −7.95, and −7.21 kcal/mol, respectively, for the monomer, and −8.05, −9.10, and −7.07 kcal/mol, respectively, for the dimer (Table 1).

HT1171 is a well-characterized inhibitor of the Mtb proteasome [13], which was used as the control compound in this study. HT1171 has been reported to bind with Thr1, Thr21, Arg19, Ser20, Val31, and Ala49 residues of Mtb proteasome [27]. Interestingly, Thr1, Ser20, and Thr21 are the common interacting residues of the Mtb proteasome with HT1171 and the selected hit compounds in this study (Figure 2a–d). BE of HT1171 against the Mtb proteasome was noted as −5.83 kcal/mol for the monomer and -5.97 kcal/mol for the dimer (Table 1).

**Figure 4:**
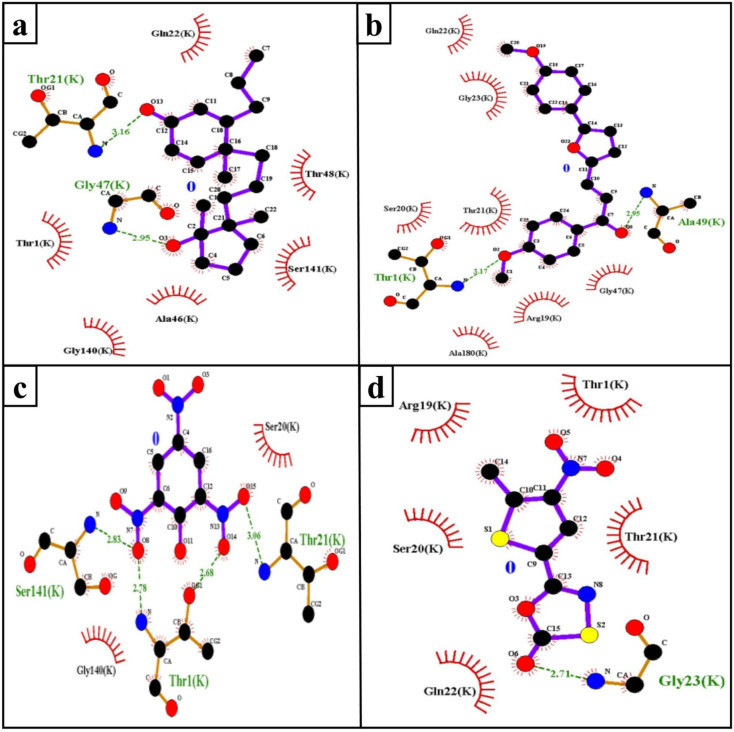
H-bond (green dashed line) and hydrophobic interacting (red arc) residues of the Mtb proteasome (monomer; K-chain) with ZINC3875469 (**a**), ZINC4076131 (**b**), ZINC1883067 (**c**), and HT1171 (**d**).

The hydrophobic interaction and H-bond help to elucidate the potency of inhibitors to the target protein and contribute an important role in “inhibitor–protein” complex stability [29,30]. The Mtb proteasome residues involved in H-bond (Table 2) and hydrophobic interaction with selected compounds are shown in Figure 4.

In the docking study, more negative BE corresponded to the strong binding of hits to the target protein. Furthermore, it is a fact that weaker binding will ultimately have a rapid dissociation rate [31]. Accordingly, in this study, the hits (ZINC3875469, ZINC4076131, and ZINC1883067) exhibited lower BE (strong binding) with the Mtb proteasome than the control compound (HT1171), suggesting that these compounds could be utilized as an inhibitor of the Mtb proteasome to combat TB. The results of the pkCSM and SwissADME tool show that top-scored compounds (ZINC3875469, ZINC4076131, and ZINC1883067) retained an acceptable range of ADMET and drug-likeness (Lipinski) (Table 3).

### 3.2. MD Simulation

MD simulations of the protein–ligand complex were performed using Gromacs 2020.4 on the Linux platform. MD simulation provides information about the receptor–ligand complex with time, so we performed the MD simulation for 20 ns on the three complexes (hit compounds). After the simulation, we analyzed the trajectory files for RMSD, RMSF, protein–ligand interactions, etc.

#### 3.2.1. RMSD

**Figure 5:**
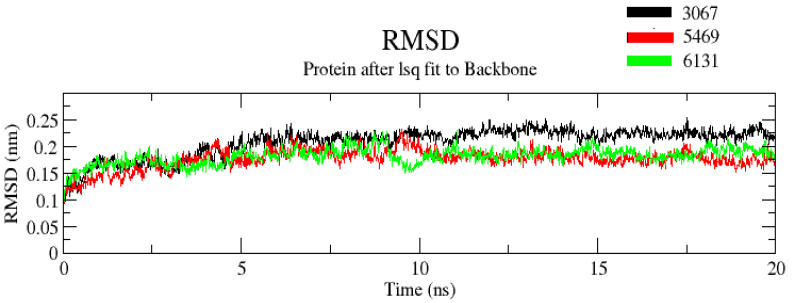
RMSD of protein and ligand after the initial RMSD values were stabilized. The RMSD graph for the backbone is shown in black color (complex ZINC1883067), red color (complex ZINC3875469), and green color (complex ZINC4076131).

The RMSD value determines the mean deviation of the complex at a specific time. It is an indicator that defines the average change in an atom’s displacement in the specific molecular conformation of the reference conformation. In trajectory analysis, the complex RMSD was found within the range of 0.25 Å. The initial RMSD value of the complex was 0.1 Å. The backbone atoms were monitored, and the stability, compactness, structural fluctuations, and protein–ligand interactions in a solvated system were examined. RMSD of the backbone was also noted to 0.2 Å (Figure 5). RMSD was found to be constant at 0.2 Å after 10 ns. The low RMSD value suggests that complexes are more stable. Moreover, it was noted that among the three complexes, complex ZINC3875469 had the lowest RMSD values. On the other hand, complex ZINC3873067 had a comparatively larger RMSD value. This suggests that complex ZINC3875469 has more stability.

#### 3.2.2. RMSF

**Figure 6:**
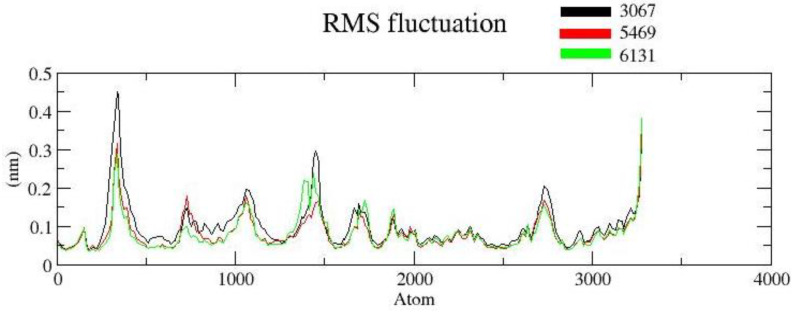
RMSF protein backbone and ligand complex is shown in black color (complex ZINC1883067), red color (complex ZINC3875469), and green color (for complex ZINC4076131).

The RMSF is useful for characterizing local protein mobility in the protein–ligand complex, which is calculated throughout the simulation. It relates to the root-mean-square displacement of each frame conformation residue relative to the average conformation used to determine the flexibility of a protein region. In an RMSF plot, the peak shows the protein area fluctuates more throughout the simulation, while the lower RMSF values reflect the less conformational transition. The atomic profile fluctuations were found to be almost similar in all three complexes. The analysis revealed that the RMSF plot (Figure 6) displayed minimal fluctuations in the protein structures for complex ZINC3875469. The protein–ligand complex displayed lower flexibility, and the RMSF plot revealed variations in certain regions of protein residues. It is suggested that the ligand binding site remained approximately rigid throughout the simulation.

#### 3.2.3. Radius of Gyration (Rg)

**Figure 7:**
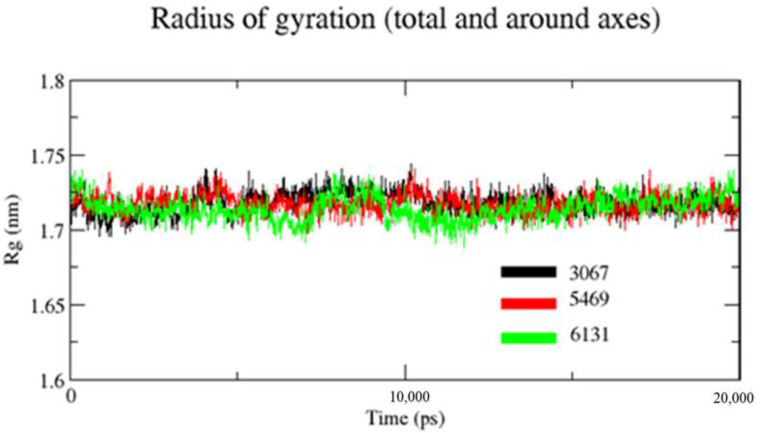
Plot for Rg of backbone atoms of the proteins in the presence of ligands. Black color (complex ZINC1883067), red color (complex ZINC3875469), and green color (complex ZINC4076131).

Rg is used to assess a characterization parameter that evaluates changes in protein structures. For the measurement of the transition in Rg of the protein backbone atoms, the gmx gyrate software was used. Figure 7 shows that the Rg values of complexes ZINC1883067, ZINC3875469, and ZINC4076131 did not change significantly throughout the simulation and continued to fluctuate at 1.73 and 1.69 nm, respectively, indicating that the ligand had little influence on protein structures. It was observed that the Rg value of complex ZINC3875469 was lower and had little fluctuation comparatively throughout the 20 ns of simulation. This suggests that the ligand–protein interaction in complex ZINC3875469 is very high, which makes its structure more compact.

#### 3.2.4. Minimum Distance

**Figure 8:**
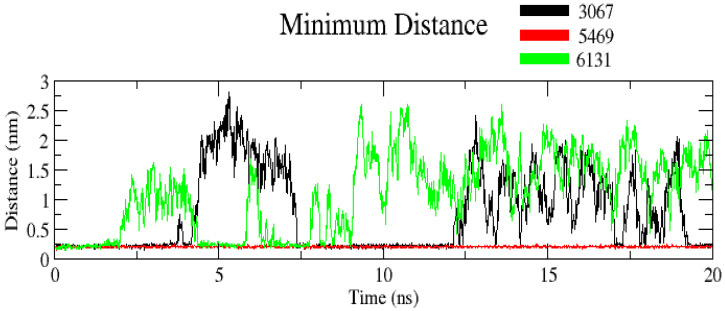
Plot showing minimum distance for complexes. Black color (complex ZINC1883067), red color (complex ZINC3875469), and green color (complex ZINC4076131).

The minimum distance between protein and ligand is given in Figure 8. The average value was found to be 1.5 nm. Interestingly, complex ZINC3875469 had the comparatively lowest minimum distance of 0.25 nm during the entire simulation, indicating more compactness and stability of complex ZINC3875469. This suggests that complex ZINC3875469 is more stable than other complexes comparatively.

#### 3.2.5. Number of Hydrogen Bonds (H-Bond Number)

**Figure 9:**
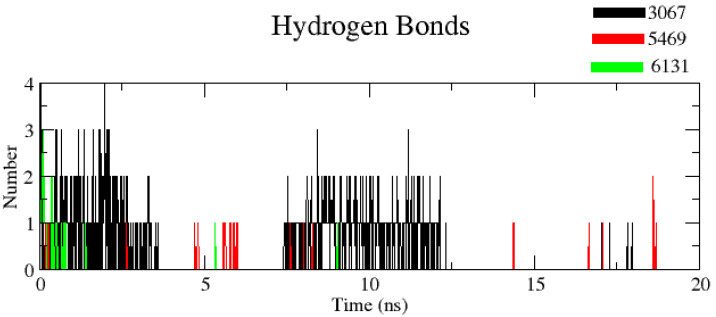
Plot showing number of hydrogen bonds for complexes. Black color (complex ZINC1883067), red color (complex ZINC3875469), and green color (complex ZINC4076131).

The number of H-bonds was measured to find out the robustness of the complex using a cut-off value 0.35 nm. It was noticed that complexes ZINC1883067 and ZINC3875469 had the most H-bonds, but the complex ZINC3875469 H-bonds were more stable over the entire simulation and thus play a significant role in stabilizing protein–ligand interactions (Figure 9).

It should be noted that the BE values and MD simulations obtained can only show the binding effectiveness and stability of inhibitors with the target protein. However, further bench work studies are required to validate these hits (ZINC3875469, ZINC4076131, and ZINC1883067) as Mtb proteasome inhibitors.

## 4. Conclusions

In summary, natural compounds from the ZINC database were screened against the Mtb proteasome. The top hit compounds (ZINC3875469, ZINC4076131, and ZINC1883067) demonstrated robust binding with the monomer as well as dimer Mtb proteasome. Molecular docking of these selected hits with the Mtb proteasome dimer model revealed that, in addition to interacting with K-chain residues, they also interacted with many other residues of the L-chain. These results open the way for further experimental confirmation in the quest for a novel Mtb proteasome inhibitor to combat TB.

## Figures and Tables

**Table 1:** Binding energy of hit compounds with Mtb proteasome (monomer and dimer).

Compound	Binding Energy (kcal/mol)		Inhibition Constant (µM)	
	Monomer	Dimer	Monomer	Dimer
ZINC3875469	−7.19	−8.05	28.9	26.54
ZINC4076131	−7.95	−9.10	43.24	0.213
ZINC1883067	−7.21	−7.07	27.92	23.96
HT1171 *	−5.83	−5.97	45.36	47.01

* Positive control for Mtb proteasome.

**Table 2:** H-bond interactions between compounds and the Mtb proteasome (monomer; K-chain).

S.No.	Target	Compound	H-Bond	H-Bond Length (Å)
**1.**	Mtb proteasome	ZINC1883067	THR1:HN3-UNK0:O8	2.78
			THR1:HG1-UNK0:O14	2.68
			THR21:HN-UNK0:O15	3.06
			SER141:HN-UNK0:O8	2.83
**2.**		ZINC4076131	THR1:HT3-UNK0:O2	3.17
			ALA49:HN-UNK0:O8	2.95
**3.**		ZINC3875469	THR21:HN-UNK0:O13	2.15
			GLY47:HN-UNK0:O3	2.49
**4.**		HT1171	GLY23:HN-UNK0:O6	2.71

**Table 3:** Pharmacokinetic properties of top-scoring ligands.

Property	Model Name		Predicted Value			Unit
			ZINC1883067	ZINC4076131	ZINC3875469	
Absorption	Water solubility		−3.67	−5.213	−4.624	log mol/L
	Caco2 permeability		−0.275	1.432	1.569	log Papp in 10–6 cm/s
	Intestinal absorption (human)		81.583	97.413	96.726	% Absorbed
	Skin Permeability		−2.786	−2.629	−2.985	log Kp
Distribution	VDss (human)		−0.44	0.315	0.397	log L/kg
	Fraction unbound (human)		0.173	0.118	0.105	Fu
	BBB permeability		−1.036	0.099	0.2	log BB
	CNS permeability		−2.744	−1.483	−2.42	log PS
Metabolism	CYP2D6 substrate		No	No	No	Yes/No
	CYP3A4 substrate		No	Yes	Yes	
	inhibitor	CYP1A2	Yes	Yes	No	
		CYP2C19	No	Yes	No	
		CYP2C9	No	Yes	No	
		CYP2D6	No	No	No	
		CYP3A4	No	No	No	
Excretion	Total Clearance		0.587	0.742	0.636	log mL/min/kg
	Renal OCT2 substrate		No	No	Yes	Yes/No
Toxicity	AMES toxicity		Yes	No	No	
	Max. tolerated dose (human)		−0.58	0.547	−0.423	log mg/kg/day
	hERG I inhibitor		No	No	No	Yes/No
	Oral Rat Acute Toxicity (LD50)		2.321	2.347	1.837	mol/kg
	Oral Rat Chronic Toxicity (LOAEL)		1.35	1.913	1.708	log mg/kg_bw/day
	Hepatotoxicity		No	No	No	Yes/No
	Skin Sensitisation		Yes	No	No	
	*T. pyriformis* toxicity		0.804	0.569	1.054	(log μg/L)
	Minnow toxicity		0.644	−2.307	0.712	
Druglikeness	Lipinski		Yes	Yes	Yes	(Yes/No)

## Data Availability

Not applicable.

## Sample Availability

Not available.
